# Titanium Application Increases Phosphorus Uptake Through Changes in Auxin Content and Root Architecture in Soybean (*Glycine Max* L.)

**DOI:** 10.3389/fpls.2021.743618

**Published:** 2021-11-11

**Authors:** Sajad Hussain, Iram Shafiq, Milan Skalicky, Marian Brestic, Anshu Rastogi, Maryam Mumtaz, Muzammil Hussain, Nasir Iqbal, Muhammad Ali Raza, Sumaira Manzoor, Weiguo Liu, Wenyu Yang

**Affiliations:** ^1^College of Agronomy, Sichuan Agricultural University, Chengdu, China; ^2^Sichuan Engineering Research Center for Crop Strip Intercropping System, Key Laboratory of Crop Ecophysiology and Farming System in Southwest China (Ministry of Agriculture), Sichuan Agricultural University, Chengdu, China; ^3^Department of Botany and Plant Physiology, Faculty of Agrobiology, Food and Natural Resources, Czech University of Life Sciences Prague, Prague, Czechia; ^4^Department of Plant Physiology, Slovak University of Agriculture, Nitra, Slovakia; ^5^Laboratory of Bioclimatology, Department of Ecology and Environmental Protection, Poznań University of Life Sciences, Poznań, Poland; ^6^State Key Laboratory of Mycology, Institute of Microbiology, Chinese Academy of Sciences, Beijing, China; ^7^School of Agriculture, Food and Wine, The University of Adelaide, Adelaide, SA, Australia; ^8^National Research Center of Intercropping, The Islamia University of Bahawalpur, Bahawalpur, Pakistan

**Keywords:** titanium, low phosphorus, root Auxin, rhizosphere pH, antioxidants

## Abstract

Phosphorus (P) is an essential macronutrient needed for plant growth, development, and production. A deficiency of P causes a severe impact on plant development and productivity. Several P-based fertilizers are being used in agriculture but limited uptake of P by the plant is still a challenge to be solved. Titanium (Ti) application increases the nutrient uptake by affecting the root growth; however, the role of Ti in plant biology, specifically its application under low light and phosphorus stress, has never been reported. Therefore, a pot study was planned with foliar application of Ti (in a different concentration ranging from 0 to 1,000 mg L^–1^) under different light and P concentrations. The result indicated that under shade and low P conditions the foliar application of Ti in different concentrations significantly improves the plant growth parameters such as root length, root surface area, root dry matter, and shoot dry matters. The increase was observed to be more than 100% in shade and low P stressed soybean root parameter with 500 mg L^–1^ of Ti treatment. Ti was observed to improve the plant growth both in high P and low P exposed plants, but the improvement was more obvious in Low P exposed plants. Auxin concentration in stressed and healthy plant roots was observed to be slightly increased with Ti application. Ti application was also observed to decrease rhizosphere soil pH and boosted the antioxidant enzymatic activities with an enhancement in photosynthetic efficiency of soybean plants under shade and P stress. With 500 mg L^–1^ of Ti treatment, the photosynthetic rate was observed to improve by 45% under shade and P stressed soybean plants. Thus, this work for the first time indicates a good potential of Ti application in the low light and P deficient agricultural fields for the purpose to improve plant growth and development parameters.

## Introduction

Macronutrients are the elements required by plants in large amounts for growth and development. Phosphorus (P) is an important macronutrient that plays a vital role in multiple biological processes, such as signaling, antioxidant reactions, chemical energy and photosynthesis, hormonal regulation as well as nucleic acid synthesis. However, at large, most of the soils contain low P, which imposes heavy P fertilizer use from synthetic sources. Globally, about 30–40% (5.7 billion hectares) of arable land is deficient in P (Zhang et al., 2019). The low availability of P is attributed to its binding with Fe^+3^ and Al^+3^ and transformation to organic matter through microbial degradation. Phosphorus deficiency suppresses plant growth and biomass accumulation. As P plays an important role in photosynthesis, its deficiency significantly affects CO_2_ metabolism in plants. Phosphorus dearth significantly reduces the efficiency of photosystem PSII and electron transport rate (ETR). Furthermore, P deficiency damages the cellular structure by the production of reactive oxygen species (ROS) such as superoxide (O_2_^–^) and hydrogen peroxide (H_2_O_2_) (Tyburski et al., 2010). P deficit conditions largely affect the root morphology-related plant hormones i.e., Auxin (IAA). To minimize the toxic effects and to maintain an optimal level of ROS, plants activate their antioxidant defense system. Moreover, plants adopt several strategies such as changing root morphology, metabolic alteration and activation of P- related genes to lower the minuscule P availability in soils.

Soybean (*Glycine max* L.) is vastly cultivated (about 667 thousand hectares) under maize-soybean relay strip intercropping in Southwestern China. However, in maize-soybean relay strip intercropping, shade (initial 35–45 days) affects the soybean growth in two ways; it affects above-ground growth by reducing plant biomass, stem strength, leaf area, net photosynthetic rate, and below-ground growth by suppressing the root length, root surface area and root volume which results in inefficient nutrient (i.e., P) uptake. Titanium (Ti), with an atomic number 22 and atomic weight of 47.88, is a transition element of Group 4 (IVB). It is the 9th most abundant element in the earth’s crust (Buettner and Valentine, 2012). It is ranked as the second most widely present transition metal (after iron [Fe]), while its elemental abundance is about 5 times < Fe and 100 times > than copper (Cu). Although Ti application in crops is very rare, but several studies reported its beneficial effects on plant growth and development. It is strongly believed that Ti has beneficial effects in plants as it accumulates in different parts i.e., roots, stems and leaves. Furthermore, the higher concentration of Ti causes phytotoxicity and decreases root growth, photosynthetic efficiency, increases ROS-induced oxidative stress and damage the DNA. Ti competes with Fe for ligands or proteins in plants when Ti concentration is high (Cigler et al., 2010). There is currently a scarcity of information on the critical levels of Ti in plant toxicity. If Ti concentration is too high (500–1,000 mg kg^–1^), it could interfere with the biological roles of Fe, resulting in Ti toxicity. Cigler et al. (2010) found that Ti at a high-level affects Fe-containing proteins and can alter the chloroplast functions.

Previous studies reported that Ti significantly enhances photosynthesis by regulating the efficiency of photosystem II (PSII) and ribulose 1,5-bisphosphate carboxylase/oxygenase (Rubisco) related genes (Tumburu et al., 2015) under an abiotic stress environment. Ti also improves tolerance to drought stress, salt stress, and low light stress (Gohari et al., 2020). At a cellular level, Ti application strengthens the effectiveness of photosynthetic apparatus by guarding the chloroplast cell structure. The latest research regarding Ti application on soybean revealed that Ti significantly improved root architecture (root length, root surface area, and root volume) under shading stress (Boykov et al., 2019; Hussain et al., 2019a). Furthermore, it is reported that Ti promotes root growth by regulating Auxin in *Arabidopsis thaliana* (Wei et al., 2020). Furthermore, Ti application increased non-structural carbohydrates of strawberry fruit suggesting that Ti regulate signaling and transport of sugar content above and below-ground parts of plant. Under abiotic stresses such as metal, drought, and salt stress, Ti enhances the scavenging of ROS by producing antioxidants such as catalase (CAT), superoxide dismutase (SOD), peroxidase (POD) (Skupień and Oszmiański, 2007). Recently, due to the rising dearth of essential nutrients in soils worldwide, the use of several elements such as Ti and silicon (Si) has received great attention in crop production. Ti has been reported to mitigate nutrient imbalance and play a dual role in deficiency and abundance (toxicity). For example, under N stress, Ti application compensated the deficiency by increasing the accumulation of other essential elements such as K, Mn, Fe, Cu, and Zn (Frutos et al., 1996; Haghighi et al., 2012). On the other hand, foliar application of Ti increases the Fe concentration in the chloroplast to boost photosynthesis. Similarly, under different concentrations of Ti, increased P uptake has been reported (Zahra et al., 2015, 2017).

In our previous findings, we observed that ionic Ti significantly improved photosynthetic efficiency, root length, root surface area, and root volume of soybean under normal light and shade conditions (Hussain et al., 2019a). These results provided us a base to investigate further the critical problem of soybean growth under relay strip intercropping i.e., inefficient root architecture (leading to poor P uptake) and reduced photosynthesis due to a low light environment. Therefore, the research was aimed for the first time to investigate the effect of various concentrations of ionic Ti on the growth, photosynthetic parameters, Rubisco enzymatic activity, antioxidants activity, chlorophyll fluorescence, root architectural changes, rhizosphere pH, root hormone Auxin (IAA), and phosphorus uptake (P) of soybean crop grown under co-occurring shade and low phosphorous stress to suggest the optimum level of ionic titanium for achieving higher photosynthetic rates and improved phosphorus uptake in the intercropping environment.

## Materials and Methods

### Titanium Ion Solution Characterization

Anti-hydrolyze stabilized ionic titanium (Paten t No. PCT/US8308840B2) was provided by Tigrow (Tianjin) Science and Technology Ltd. The formulation used in this study contains no other metallic elements and amino acids, and the stock concentration of titanium was 4,000 mg L^–1^ (Li et al., 2018).

### Plant Material and Experimental Layout

The experiment was conducted in the modern agricultural research and development base of Sichuan Agricultural University in Chongzhou city, Sichuan Province, China. A complete randomized design was used for the experiment. Five to seven seeds of Nandou 12 (soybean cultivar) were planted in plant pots (30 cm in diameter and 20 cm high). Each treatment contained 5 pots with five replications. The shading condition was provided by a black shade net with 28% of full sunlight. On the eighth day after emergence, different concentrations of Ti (T0 = 0, T1 = 125, T2 = 250, T3 = 500 and T4 = 1,000 mg L^–1^) were foliar applied on soybean seedlings grown in high phosphorus (HP = 100 mg kg^–1^) and low phosphorus (LP = 10 mg kg^–1^) concentrations in the pots under normal light (NL) and shade (SC) conditions. This foliar application was done two times with an interval of 8 days. The soybean seedlings were grown for 35 days under the above-mentioned conditions. The latest fully expanded leaves were then chosen for physiological measurements after one month.

### Agronomic Traits

Plant height, stem diameter, and dry weights were taken after one month. The samples were packed in an envelope and placed in an oven at 105°C for 40 minutes and then at 85°C for 4 days. Plant height and stem diameter was measured using meter scale and vernier caliper, respectively. Dry weight was measured using an electronic balance.

### Chlorophyll Fluorescence

Chlorophyll fluorescence parameters were determined by using the destructive sampling method. The latest fully expanded leaves of each treatment were selected. Fluor Imager softer was used to investigate the fluorescence parameters. The protocol followed has been described in our previous publications (Hussain et al., 2019a,b).

### Photosynthetic Rate

Photosynthetic rate and stomatal conductance were measured by using portable photosynthetic meter LICOR-6400 (LI-6400-09; LiCor, Lincoln, NB, United States). The measurements were taken on a full sunny day. Manual control conditions were as follows; CO_2_ concentration 400 μmoL mL^–1^ and light intensity 450 μmoL m^–2^s^–1^ (Iqbal et al., 2019).

### Analysis of Rubisco Enzyme

The activity of RuBP carboxylase in the latest fully expanded leaves of soybean was measured using assay kits by following the manufacturer’s instructions (Plant RuBP carboxylase kit, Wuhan Purity Biological Technology Co. Ltd, China). The leaves samples were destructively taken from all treatments and immediately stored in liquid nitrogen. 1 g of frozen leaf samples of each treatment were ground using 2 ml of 50 mmol L^–1^ phosphate buffer solution (pH 7.8) with the help of mortar and pestle in an icebox. The solution was then centrifuged at 4°C for 15 min at 7,000 rcf, the micropore plate encapsulated the rubisco activase antibody to form a solid phase antibody. This was added to the micropore of the monoclonal antibody. Then a phosphate buffer solution (40 μl) was added first as a buffer solution in the micropore plate, then 10 ml of sample solution was added. The micropore plate was then sealed by a plastic film and incubated at 37°C for 30 min. The incubation was repeated over five times. The 3,3′5,5′-tetramethylbenzidine was transferred under the catalysis of horseradish peroxidase enzyme, which first turned blue and finally to a yellow color under the action of an acid. The stop solution was added, and absorbance was measured within 15 min at 450 nm wavelength by an enzyme marker. Then, a standard curve was drawn and RubisCO activity was expressed as Ug-1 (Hussain et al., 2019a).

### Enzymatic Activities

The latest fully expanded leaves were chosen and immediately stored at −80°C by using liquid nitrogen. 0.15 g of each leaf sample was then ground with 1.5 mL of ice-cold 50 mM HEPES buffer (pH 7.8) containing 0.2 mM EDTA, 2 mM ascorbic acid (AsA), and 2% PVP (w/v). Homogenates were centrifuged at 12,000*g* for 20 min at 4°C. The obtained supernatants were then used for the spectrophotometric determination of enzymatic activity of Superoxide dismutase (SOD), Peroxidase (POD), Catalase (CAT), and MDA activity according to the method (Hussain et al., 2020).

### Root Morphology Assessment

Soybean plants were uprooted after 30 days gently and placed in running water to remove the soil. Root morphological traits including root length, surface area, volume and diameter were determined as described in our previous studies (Hussain et al., 2019a). The roots were placed on a glass plate containing minuscule amount of water. The roots were spread carefully on a plexiglass plate and transferred to the root Epson perfection V700 photo scanner. After scanning, the obtained root images were analyzed for root architectural measurements by WinRhizo software (Version 2007d, Regent Instrument Inc., Canada).

### Rhizosphere pH

The rhizosphere soil and soil adhered to roots were gently separated. The soil was air-dried and ground. After that, the soil (5 g) and distilled water (25 ml) were added in a 50 ml beaker. The solution was stirred for 30 min. To determine the pH, an electric pH meter was used (Zahra et al., 2019).

### Root Auxin Content

The samples for root Auxin were taken at the V4 stage and immediately placed in liquid nitrogen. Six samples were collected from each treatment, and all samples were stored at −80°C. Auxin content was measured by following the manufacturer’s instructions (ELISA of plant hormones assay kit, Shanghai Enzyme-linked Biotechnology Co., Ltd.) (Li et al., 2019).

### Statistical Analyses

The properties of studied plant soybeans in individual populations were compared using Three-Way ANOVA and Tukey’s *post hoc* test in the Statistix 8.1. Program Canoco 5 (Lepš and Šmilauer, 2003) was used for PCA (Principal Component Analysis) calculated from centered data. This analysis was used to figure out the relationship among Ti under NL and SC combined with low phosphorus (LP) and high phosphorus (HP) stress. PCA is a multivariate statistical analysis to find the relationship among dependent and independent variables.

## Results

### Agronomic Traits

**FIGURE 1:**
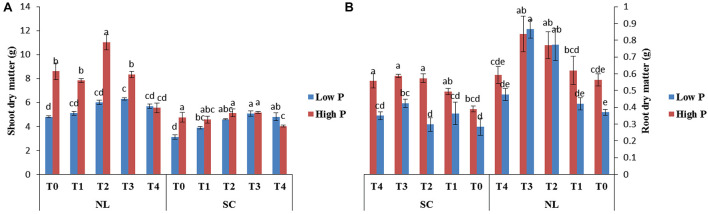
Effect of Ti on **(A)** shoot dry matter and **(B)** root dry matter of soybean under NL and SC combined with LP and HP conditions. The bar plots with different lettering show the significant difference among cultivars at probability < 0.05. The error bars are the ± SD values with *n* = 3.

Figure 1 shows the effect of titanium (Ti) treatments (T0, T1, T2, T3, and T4 respectively) on soybean shoot and root dry matter in response to low (LP) and high phosphorous (HP) under shade (SC) and normal light (NL) conditions while Table 1 shows the possible interaction of environment (SC, NL), phosphorous treatments (LP, HP) in combination with Ti application The ANOVA of root and shoot dry matter reveals the significant interaction (*P* < 0.05) of all factors.

**TABLE 1:** Values under ANOVA are the F-test, probabilities (*P* value) and coefficient of variation of the sources of variation (LSD, *P* < 0.05).

ANOVA				

Factors	Root dry matter		Shoot dry matter	
Phosphorus (P)	*F* = 90.54	*P* = 0.0000	*F* = 294.44	*P* = 0.0000
Environment (Env)	*F* = 201.98	*P* = 0.0000	*F* = 718.98	*P* = 0.0000
Treatments (Treat)	*F* = 44.12	*P* = 0.0000	*F* = 47.78	*P* = 0.0000
P*Env	*F* = 7.05	*P* = 0.0115	*F* = 163.45	*P* = 0.0000
P*Treat	*F* = 1.72	*P* = 0.1654	*F* = 45.25	*P* = 0.0000
Env*Treat	*F* = 15.00	*P* = 0.0000	*F* = 20.57	*P* = 0.0000
P*Env*Treat	*F* = 6.50	*P* = 0.0004	*F* = 11.71	*P* = 0.0000
CV	10.49		6.01	

Under NL, shoot dry matter was significantly higher in HP as compared to LP except for treatment T4 where the difference was non–significant (Figure 1A). The maximum value in NL was observed in T2 (11.03 g) under HP whereas T3 significantly improved (23.81%) shoot dry matter under LP in comparison to control. Under SC, Ti treatments significantly improved shoot dry matter in LP as compared to control, with maximum value observed in T3 (5.06 g). In T3, the difference between the values recorded in HP and LP became non-significant whereas the values exceeded significantly in LP (20.36%) in comparison to HP in T4.

**FIGURE 2:**
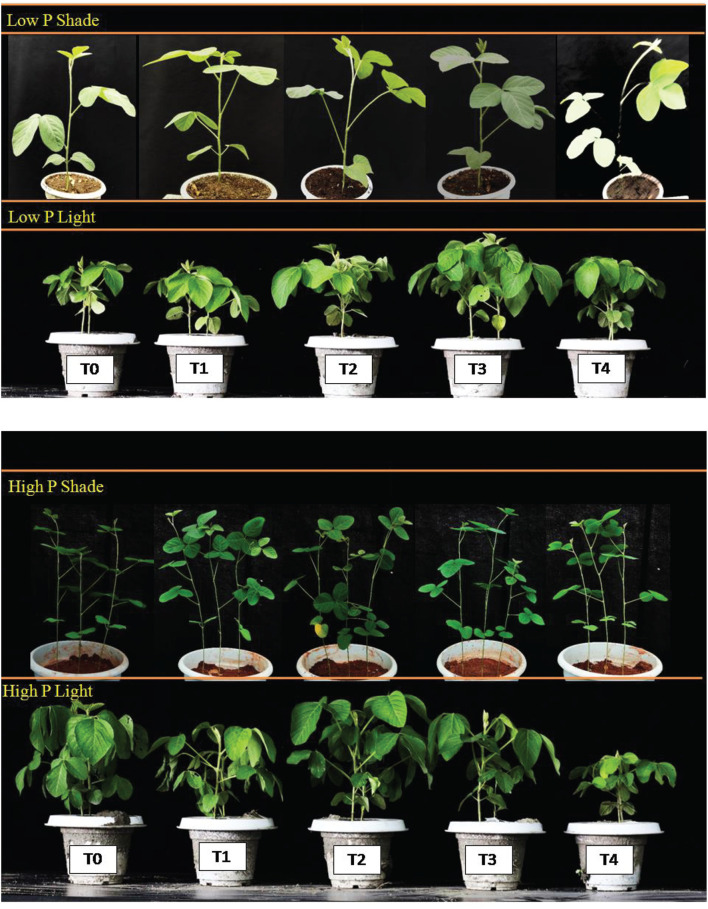
Phenotypic demonstration of soybean plants under variable phosphorus (HP, LP) and light environment (NL, SC).

Under NL, root dry matter increased significantly in response to T2 and T3 as compared to T0 in HP and LP (Figure 1B). However, the difference in root dry matter values between HP and LP against various Ti treatments remained non-significant, the maximum value was recorded in T3 (0.866 g) under LP. Under SC, a significant increase in root dry mater values was observed in T2 (31.58%), T3 (32.76%) and T4 (29.09%) in comparison to T0 under HP with the maximum value recorded in T3 (0.586 g) whereas in LP, the values increased significantly in response to T3 (33.01%) as compared to T0. The phenotypic real-time representation confirms the obvious changes in plant growth (Figure 2).

### Chlorophyll Fluorescence

**FIGURE 3:**
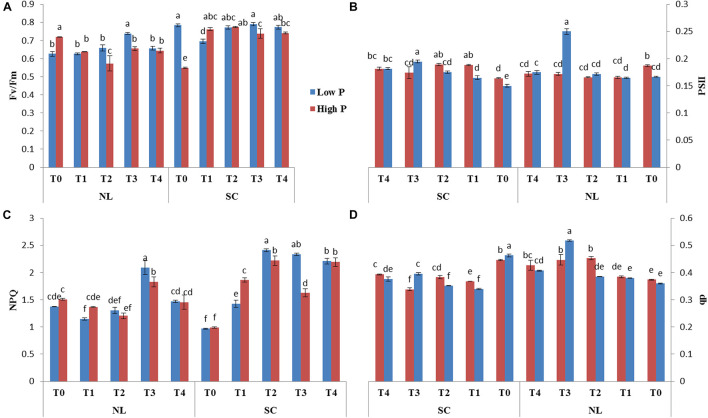
Effect of Ti on **(A)** Fv/Fm, **(B)** PSII, **(C)** NPQ, and **(D)** qp of soybean leaf under NL and SC combined with LP and HP conditions. The bar plots with different lettering show the significant difference among cultivars at probability < 0.05. The error bars are the ± SD values with *n* = 3.

Chlorophyll fluorescence parameters (Fv/Fm, PSII, NPQ and qP) were influenced by variable light environment, phosphorus levels and Ti treatments (Figure 3). Under NL, in comparison to control (T0), T1, T2, T3, and T4 resulted in a significant decrease (12.63, 25.54, 9.83, and 11.77%, respectively) in Fv/Fm values in HP. In LP, Fv/Fm values did not change significantly in response to Ti treatments as compared to T0 except for T3 which resulted in a significant increase (15.12%) against control (Figure 3A). Under SC, in comparison to T0, T1, T2, T3, and T4 resulted in a significant increase (28.11, 29.23, 25.78, and 26.05%, respectively) in Fv/Fm values in HP with the maximum value recorded in T2 (0.774). In LP, Fv/Fm decreased significantly (12.65%) in T1 as compared to control whereas in T2, T3, and T4, the difference with the control remained non-significant. Overall, under SC, the maximum Fv/Fm value was recorded in T0LP (0.783) while the minimum value was recorded in T0HP (0.548).

Under NL, PSII decreased significantly in response to T1, T2, T3, and T4 (12.88, 12.86, 8.93, and 8.72% respectively) as compared to control in HP, however, the difference among T1, T2, T3, and T4 values remained non-significant (Figure 3B). In LP, a significant increase (33.33%) in PSII was observed in response to T3 as compared to T0 while the values of remaining treatments did not differ significantly from the control. Contrasting to NL, under SC, a significant increase in PSII was observed in response to Ti treatments as compared to control (except for T3 where the difference was non-significant) in HP. Similarly, in LP, PSII increased with increasing concentration of Ti compared to control. Overall, under SC, the maximum value was observed in T3LP (0.194) whereas a minimum value was recorded in T1LP (0.15).

Under NL, NPQ decreased significantly in T2 (24.91%) whereas it increased significantly in T3 (17.62%) as compared to control in HP. In LP, a significant decrease (19.71%) in NPQ was observed in response to T1 against control while similar to T3 in HP, NPQ also increased significantly (34.35%) in LP in response to T3. Contrary to NL, under SC, NPQ increased significantly in response to increasing Ti concentration in HP with maximum value observed in T2 (2.22). Similarly, in LP, increasing Ti concentration increased the NPQ values with maximum increase recorded in T2 (2.411). Overall, in SC, maximum (2.411) and minimum (0.96) NPQ values were recorded in T2LP and T0LP, respectively (Figure 3C).

**FIGURE 4:**
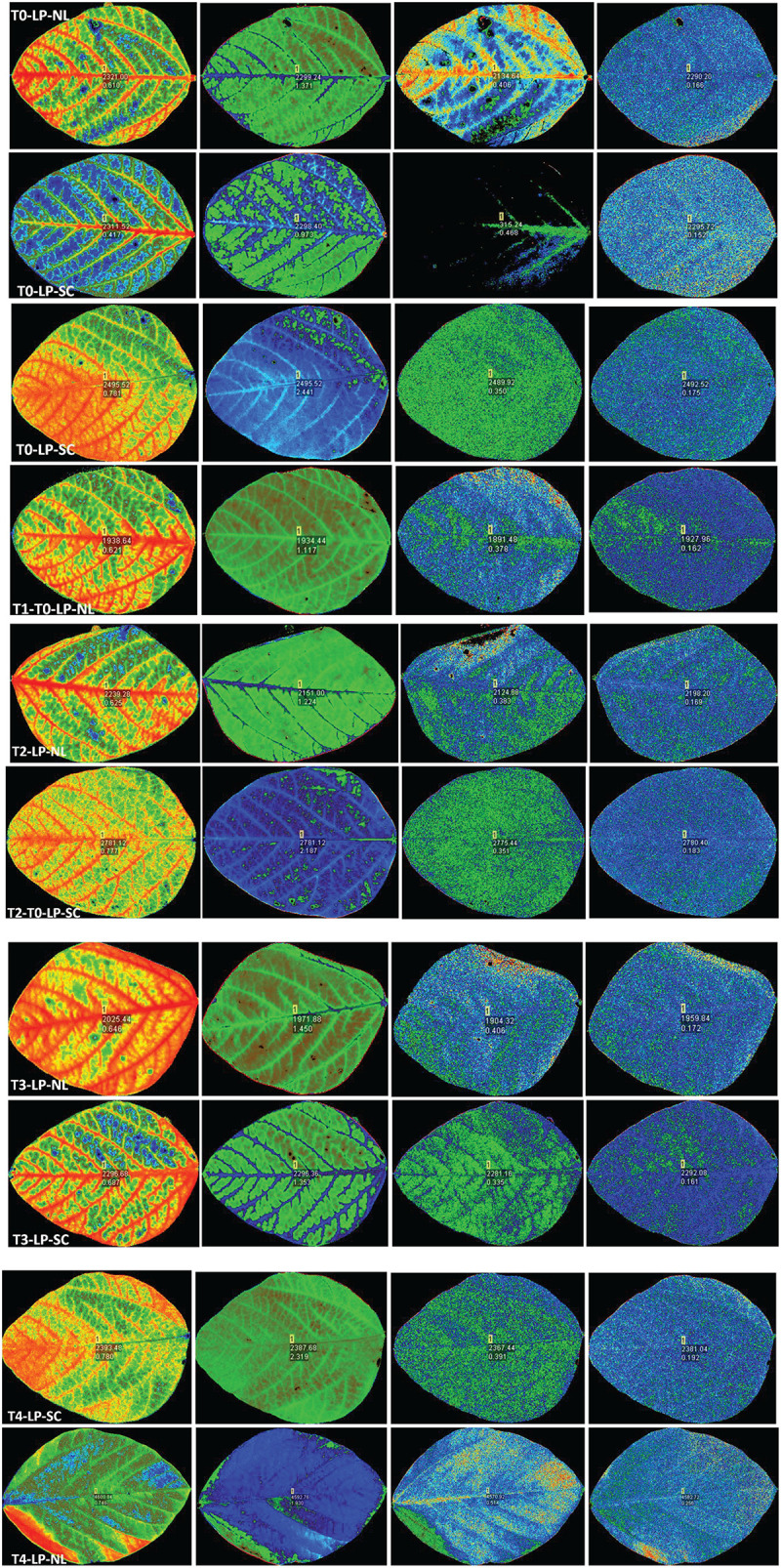
Leaf chlorophyll fluorescence of soybean in response to Ti application under NL and SC combined with LP conditions.

**FIGURE 5:**
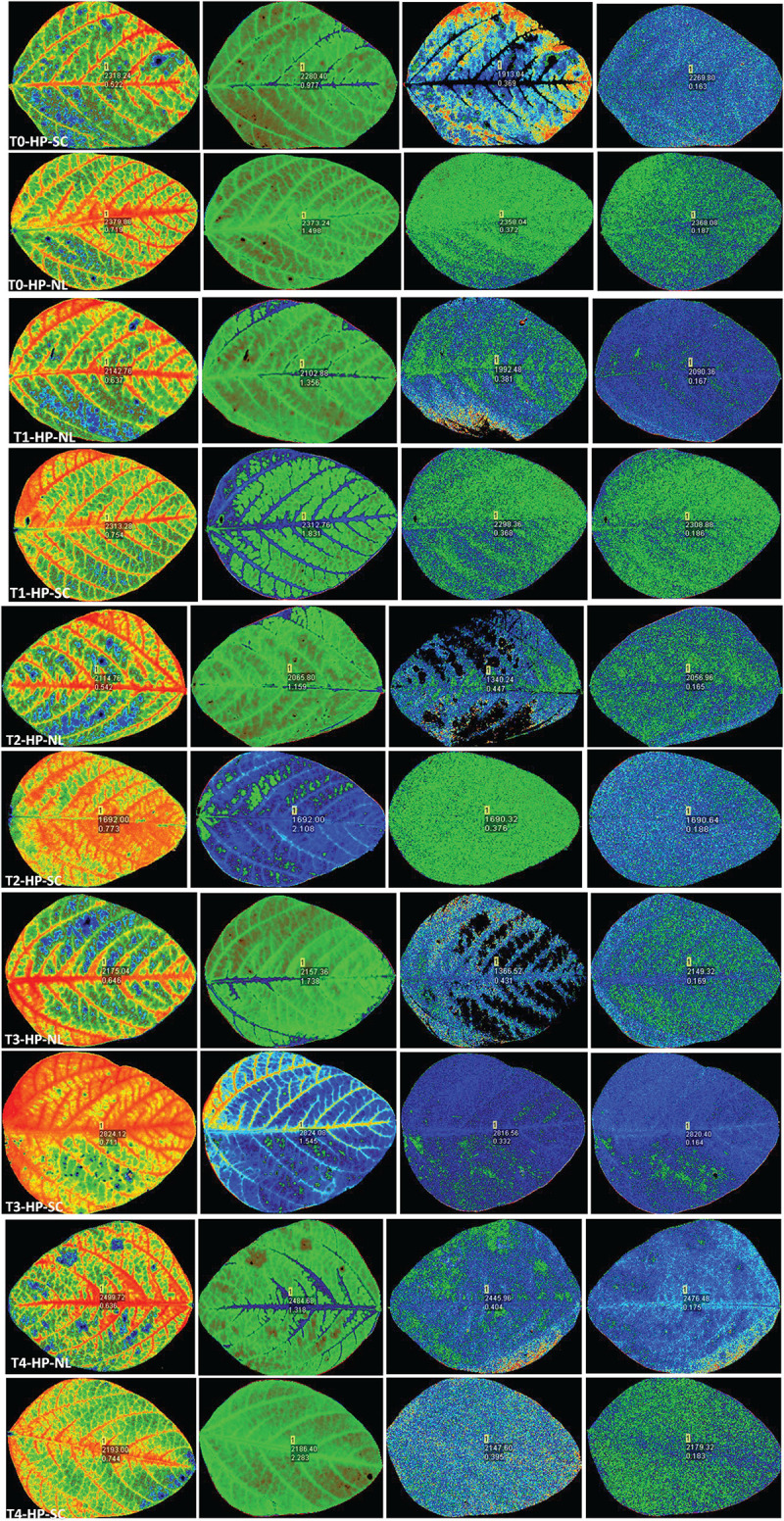
Leaf chlorophyll fluorescence of soybean in response to Ti application under NL and SC combined with HP.

qP increased significantly in response to T2 (17.45%), T3 (16.41%) and T4 (12.63%) against control in HP under NL with the maximum value recorded in T2 (0.452) whereas in LP, qP increased significantly in response to T3 (30.35%) and T4 (11.61%) as compared to control (Figure 3D). Overall, in NL, maximum (0.517) and minimum (0.360) qP values were recorded in T3LP and T0LP, respectively. Under SC, in HP, qP decreased significantly with increasing Ti concentration with the minimum value recorded in T3 (0.338). Similarly, in LP, significant decrease in qP values in response to increasing Ti concentrations was observed where minimum value was recorded in T1 (0.339). The real time leaf chlorophyll fluorescence was examined with Fluor Imager software (Figures 4, 5). Table 2 shows the possible interaction of environment (SC, NL), phosphorous treatments (LP, HP) in combination with Ti application for chlorophyll fluorescence parameters.

**TABLE 2:** Values under ANOVA are the F-test, probabilities (*P* value) and coefficient of variation of the sources of variation (LSD, *P* < 0.05).

**ANOVA**								

**Factors**	**Fv/Fm**		**PSII**		**NPQ**		**qP**	
Phosphorus (P)	*F* = 81.95	*P* = 0.0000	*F* = 11.71	*P* = 0.0015	*F* = 9.20	*P* = 0.0043	*F* = 4.64	*P* = 0.0377
Environment (Env)	*F* = 533.77	*P* = 0.0000	*F* = 6.89	*P* = 0.0124	*F* = 465.95	*P* = 0.0000	*F* = 229.33	*P* = 0.0000
Treatments (Treat)	*F* = 33.08	*P* = 0.0000	*F* = 112.54	*P* = 0.0000	*F* = 297.28	*P* = 0.0000	*F* = 104.38	*P* = 0.0000
P*Env	*F* = 22.14	*P* = 0.0000	*F* = 90.93	*P* = 0.0000	*F* = 7.24	*P* = 0.0000	*F* = 2.84	*P* = 0.0999
P*Treat	*F* = 30.60	*P* = 0.0000	*F* = 141.93	*P* = 0.0000	*F* = 69.11	*P* = 0.0000	*F* = 102.58	*P* = 0.0000
Env*Treat	*F* = 54.91	*P* = 0.0000	*F* = 71.77	*P* = 0.0000	*F* = 272.84	*P* = 0.0000	*F* = 312.19	*P* = 0.0000
P*Env*Treat	*F* = 108.20	*P* = 0.0000	*F* = 29.97	*P* = 0.0000	*F* = 10.98	*P* = 0.0000	*F* = 10.00	*P* = 0.0000
CV	2.02		2.19		3.79		1.80	

### Photosynthetic Rate

**FIGURE 6:**
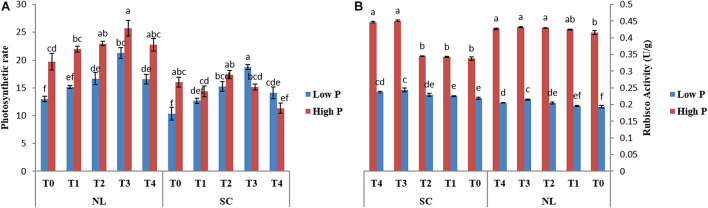
Effect of Ti application on **(A)** photosynthetic rate and **(B)** Rubisco activity of soybean leaf under NL and SC combined with LP and HP conditions. The bar plots with different lettering show the significant difference among cultivars at probability < 0.05. The error bars are the ± SD values with *n* = 3.

Under NL, Pn increased significantly in response to T2 (14.06%) and T3 (30.46%) against T0 (control) in HP whereas in LP, a significant increase was recorded in T2 (21.98%), T3 (38.81%), and T4 (21.43%) as compared to control (Figure 6A). Overall, in NL, maximum (25.74 μmol (CO_2_) m^–2^s^–1^) and minimum (13.003 μmol (CO_2_) m^–2^s^–1^) Pn values were recorded in T3HP and T0LP, respectively. Under SC, no significant difference was observed in T1, T2, and T3 Pn values as compared to T0 whereas a significant decrease (41.48%) was recorded in T4. In LP, T2 and T3 resulted in a significant increase (32.28 and 44.78%), respectively) in Pn values in comparison to control. Overall, in SC, maximum (18.73 μmol (CO_2_) m^–2^s^–1^) and minimum (10.337 μmol (CO_2_) m^–2^s^–1^) Pn values were recorded in T3LP and T0LP, respectively. Table 3 shows the possible interaction of environment (SC, NL) ^∗^ phosphorous treatments (LP, HP) in combination with Ti application for photosynthetic rate.

**TABLE 3:** Values under ANOVA are the F-test, probabilities (*P* value) and coefficient of variation of the sources of variation (LSD, *P* < 0.05).

**ANOVA**				

**Factors**	**Photosynthetic rate**		**Rubisco**	
Phosphorus (P)	*F* = 169.95	*P* = 0.0000	*F* = 47454.0	*P* = 0.0000
Environment (Env)	*F* = 378.82	*P* = 0.0000	*F* = 54.13	*P* = 0.0000
Treatments (Treat)	*F* = 53.88	*P* = 0.0000	*F* = 423.58	*P* = 0.0000
P*Env	*F* = 112.25	*P* = 0.0000	*F* = 1608.09	*P* = 0.0000
P*Treat	*F* = 15.50	*P* = 0.0000	*F* = 144.54	*P* = 0.0000
Env*Treat	*F* = 8.72	*P* = 0.0000	*F* = 223.22	*P* = 0.0000
P*Env*Treat	*F* = 7.53	*P* = 0.0001	*F* = 189.28	*P* = 0.0000
CV	5.88		1.08	

### Analysis of Rubisco Enzyme

Under NL, RA increased significantly in T2 (3.33%), T3 (3.706%), and T4 (2.65%) as compared to control in HP, though the difference among T2, T3, and T4 values remained non-significant (Figure 6B). In LP, a significant increase in RA was observed in response to T2 (5.53%), T3 (10.11%), and T4 (5.85%) as compared to control. Overall, in NL, maximum (0.4317 U/g) and minimum (0.193 U/g) values were recorded in T3HP and T0LP, respectively. Under SC, in HP, RA increased significantly only in response to T3 (25.10%) and T4 (24.33%) as compared to T0 whereas in LP, it increased significantly in T2 (4.37%), T3 (10.24%), and T4 (7.64%), respectively against T0. Overall, in SC, maximum (0.4517 U/g) and minimum (0.219 U/g) RA values were recorded in T3HP and T0LP, respectively. Table 3 shows the possible interaction of environment (SC, NL), phosphorous treatments (LP, HP) in combination with Ti application for Rubisco enzyme activity.

### Enzymatic Activities

**FIGURE 7:**
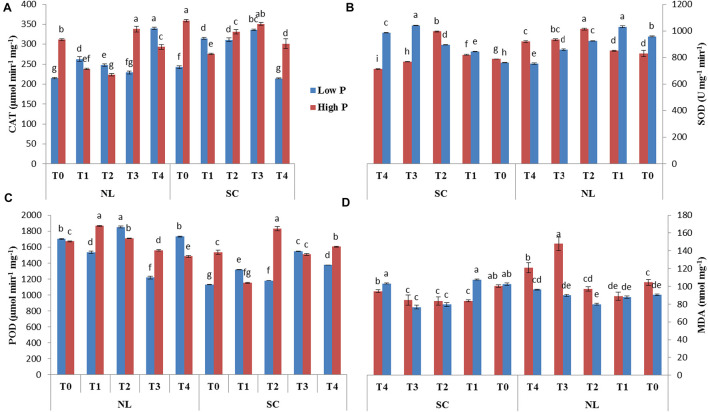
Effect of Ti application on **(A)** Catalase (CAT), **(B)** Superoxide dismutase (SOD), **(C)** Peroxidase (POD), and **(D)** Malondialdehyde (MDA) activity of soybean leaf under NL and SC combined with LP and HP conditions. The bar plots with different lettering show the significant difference among cultivars at probability < 0.05. The error bars are the ± SD values with *n* = 3.

The antioxidant enzymatic activities (SOD, POD, CAT, and MDA) were significantly affected by foliar application of Ti under combined phosphorus and light stress (Figure 7). Under NL, CAT activity in HP decreased in response to T1 (17.37%), and T2 (7.76%) significantly as compared to control whereas it increased significantly in response to T3 (29.37%). Contrarily, in LP, a significant increase in CAT activity was recorded in T1 (18.02%) and T2 (13.13%) against T0, the difference of T3 with T0 remained non-significant while the maximum increase was observed in response to T4 (339.8). Under SC, in HP, CAT activity decreased in response to T1 (30.41%), T2 (8.5%), and T4 (19.24%) as compared to control, the difference of T3 with T0 remained non-significant. Overall, under SC, maximum (359.47) and minimum (242.53) values were recorded in T0HP and T0LP, respectively (Figure 7A).

Under NL, SOD activity increased significantly in response to T2 (18.07%), T3 (11.12%), and T4 (9.75%) as compared to T0 in HP where maximum value was recorded in response to T2 (1013.86). Contrarily, in LP, SOD activity only increased significantly in response to T1 (6.99%) while it decreased significantly in T2 (3.62%), T3 (11.51%), and T4 (27.19%), respectively in comparison to control. Overall, under NL, the maximum (1031.00) and minimum (753.95) values were recorded in T1LP and T4LP, respectively. Under SC, T1 and T2 resulted in a significant increase (3.91 and 20.75%, respectively) in SOD activity in HP against control whereas in response to T3 and T4, the values decreased by 2.72 and 10.68%, respectively against T0. In LP, all Ti treatments led to a significant increase in SOD activity with the maximum value (1041.915) observed in T3 and minimum value (761.107) observed in T0 (Figure 7B).

Under NL, POD activity increased significantly in T1 (10.45%) and T2 (2.36%) and T4 (3.59%) in comparison to control in HP. In LP, POD activity increased significantly only in response to T2 (7.94%) whereas it decreased significantly in T1 (10.81%) and T3 (39.91%) against T0. Overall, under NL, the maximum (1867.466) and minimum (1217.963) values were recorded in T1HP and T3LP, respectively. Under SC, a significant increase in POD values was observed only in T2 (16.28%) and T4 (4.43%) against T0 in HP whereas in LP, all treatments led to a significant increase in PD values in comparison to T0 with a maximum (1547.216) and minimum (1129.064) value recorded in T3 and T0, respectively (Figure 7C).

Under NL, MDA increased significantly in response to T3 (41.59%) and T4 (13.67%) against T0 in HP whereas in LP, the difference of all treatments (T1; 87.83, T2; 79.89, T3; 89.7 and T4; 96.29) with T0 (90.35) was non-significant. Overall, under NL, maximum (120.98) and minimum (79.89) values were recorded in T4HP and T2LP, respectively. Under SC, in HP, MDA values decreased significantly in T1 (19.94%), T2 (20.22%), and T3 (18.79%) in comparison to T0 whereas in LP, a significant decrease was recorded in T2 (29.04%) and T3 (34.01%) in comparison to control (Figure 7D). Table 4 shows the possible interaction of environment (SC, NL) phosphorous treatments (LP, HP) in combination with Ti application for enzymatic activities.

**TABLE 4:** Values under ANOVA are the F-test, probabilities (*P* value) and coefficient of variation of the sources of variation (LSD, *P* < 0.05).

**ANOVA**								

**Factors**	**CAT**		**SOD**		**POD**		**MDA**	
Phosphorus (P)	*F* = 518.61	*P* = 0.0000	*F* = 458.72	*P* = 0.0000	*F* = 1772.77	*P* = 0.0000	*F* = 104.98	*P* = 0.0000
Environment (Env)	*F* = 621.22	*P* = 0.0000	*F* = 556.53	*P* = 0.0000	*F* = 4628.56	*P* = 0.0000	*F* = 95.62	*P* = 0.0000
Treatments (Treat)	*F* = 109.40	*P* = 0.0000	*F* = 510.93	*P* = 0.0000	*F* = 448.06	*P* = 0.0000	*F* = 55.37	*P* = 0.0000
P*Env	*F* = 43.48	*P* = 0.0000	*F* = 558.77	*P* = 0.0000	*F* = 686.83	*P* = 0.0000	*F* = 236.78	*P* = 0.0000
P*Treat	*F* = 320.05	*P* = 0.0000	*F* = 336.42	*P* = 0.0000	*F* = 205.28	*P* = 0.0000	*F* = 62.47	*P* = 0.0000
Env*Treat	*F* = 326.64	*P* = 0.0000	*F* = 210.87	*P* = 0.0000	*F* = 1098.69	*P* = 0.0000	*F* = 80.35	*P* = 0.0000
P*Env*Treat	*F* = 188.46	*P* = 0.0000	*F* = 1046.61	*P* = 0.0000	*F* = 1611.18	*P* = 0.0000	*F* = 13.84	*P* = 0.0000
CV	1.83		0.86		0.81		3.63	

### Root Morphology Assessment

**FIGURE 8:**
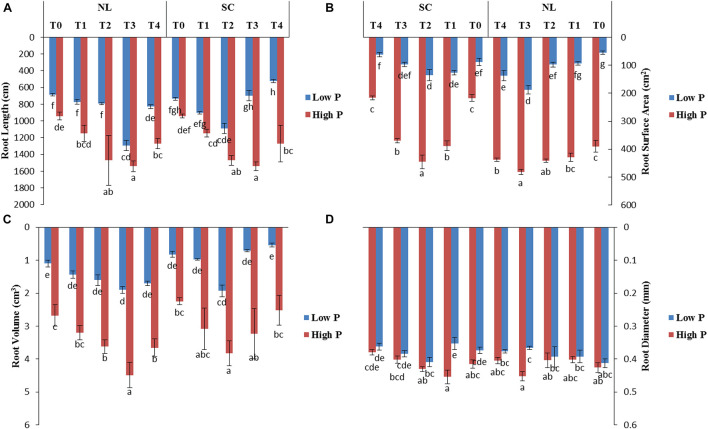
Effect of Ti application on **(A)** root length, **(B)** root surface area, **(C)** root volume, and **(D)** root diameter of soybean under NL and SC combined with LP and HP conditions. The bar plots with different lettering show the significant difference among cultivars at probability < 0.05. The error bars are the ± SD values with *n* = 3.

**FIGURE 9:**
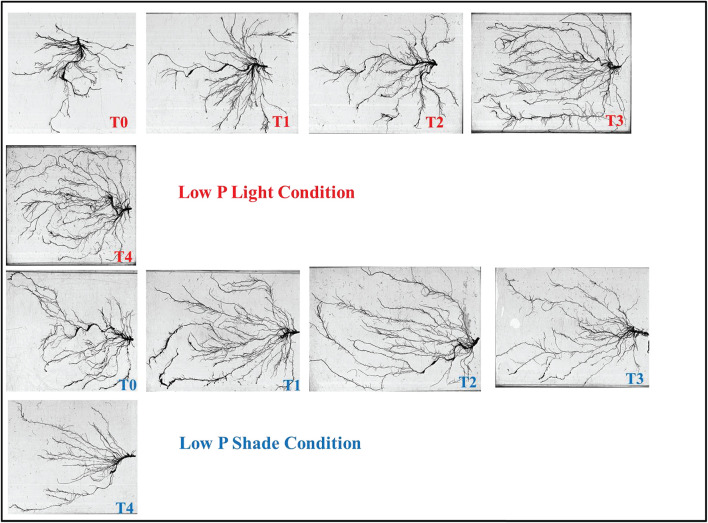
Effect of Ti application on root morphology of the soybean under NL and SC combined with LP conditions.

**FIGURE 10:**
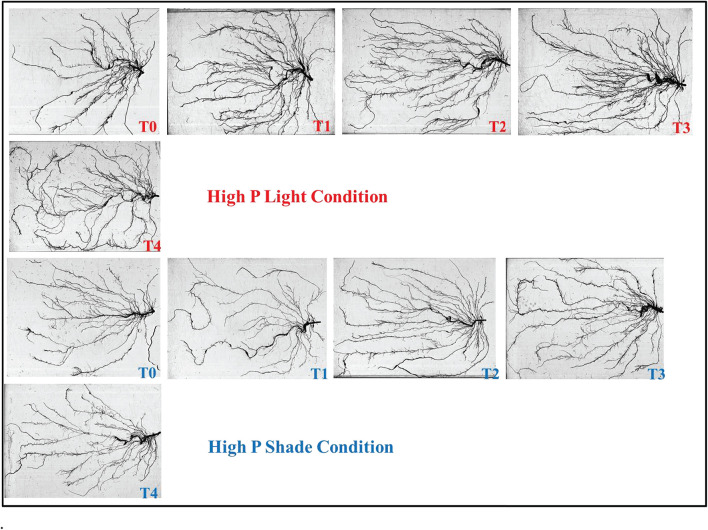
Effect of Ti application on root morphology of the soybean under NL and SC combined with HP conditions.

**FIGURE 11:**
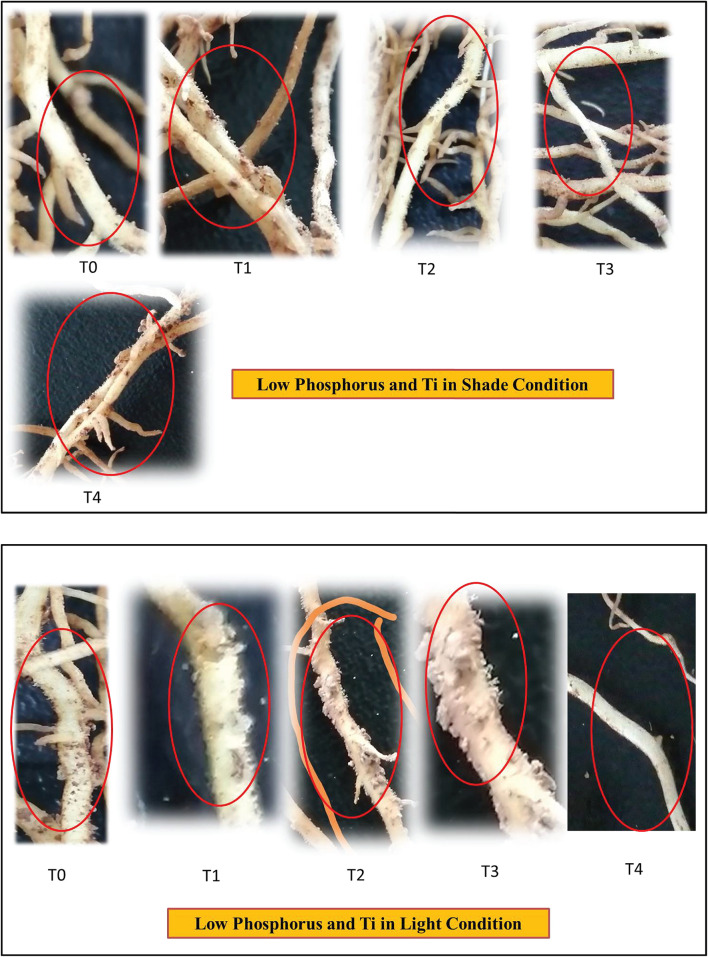
Effect of Ti on root hair of soybean under NL and SC combined with LP conditions.

Root architecture of the soybean plants was significantly affected by the application of Ti under light and phosphorus stress (Figures 8–11). Under NL, increasing the Ti concentration significantly increased the root length in comparison to T0 in HP. Maximum (1541.1 cm) and minimum (941.8 cm) values were recorded in T3 and T0, respectively whereas in LP, T3 (1294.6 cm) and T4 (829.66 cm) resulted in a significant increase in root length against control (686.7 cm). Under SC, T2 (35.97%), T3 (38.89%), and T4 (25.93%) significantly increased root length against T0 in HP while in LP, only T3 led to a significant increase (32.60%) in root length in comparison to control. Overall, under SC, maximum (1271.57 cm) and minimum (696.3 cm) values were recorded in T4HP and T3LP, respectively (Figure 8A).

Root surface area and root volume were generally significantly higher in HP as compared to LP under both the environments; NL and SC. More specifically, under NL, T3 (481.5 cm^2^ and 188.05 cm^2^) led to maximum root surface area in HP and LP, respectively. Under SC, in HP, T1 (44.06%, T2 (51.29%), and T3 (41.24%) lead to a significant increase in root surface area against T0 while in LP, only T2 led to a significant increase (35.13%) in root surface area in comparison to control (Figure 8B). Under NL, root volume was recorded maximum (4.49 cm^3^) and minimum (1.1003 cm^3^) in T3HP and T0LP, respectively while under SC, T2 significantly improved root volume (26.12%) against T0 in HP whereas in LP, the difference among all treatments remained non-significant except T4 which led to a significant reduction (51.31%) in root volume as compared to control (Figure 8C).

Under NL, no significant difference in root diameter values was observed in response to Ti treatments in HP and LP against T0, except for treatment T3 which led to a significant increase (5.81% in HP. Similar to NL, under SC, no significant difference in root diameter values was observed in response to Ti treatments in HP and LP against T0. Overall, under SC, maximum (0.4542 mm) and minimum (0.3533 mm) values were recorded in T1HP and T1LP, respectively (Figure 8D). Table 5 shows the possible interaction of environment (SC, NL), phosphorous treatments (LP, HP) in combination with Ti application for root architecture.

**TABLE 5:** Values under ANOVA are the F-test, probabilities (*P* value) and coefficient of variation of the sources of variation (LSD, *P* < 0.05).

**ANOVA**								

**Factors**	**Root length**		**Root surface area**		**Root volume**		**Root diameter**	
Phosphorus (P)	*F* = 497.15	*P* = 0.0000	*F* = 4756.49	*P* = 0.0000	*F* = 487.86	*P* = 0.0000	*F* = 62.39	*P* = 0.0000
Environment (Env)	*F* = 56.74	*P* = 0.0000	*F* = 232.51	*P* = 0.0000	*F* = 37.69	*P* = 0.0000	*F* = 2.35	*P* = 0.1333
Treatments (Treat)	*F* = 41.75	*P* = 0.0000	*F* = 92.36	*P* = 0.0000	*F* = 16.68	*P* = 0.0000	*F* = 7.17	*P* = 0.0002
P*Env	*F* = 18.93	*P* = 0.0000	*F* = 146.74	*P* = 0.0000	*F* = 0.00	*P* = 0.9936	*F* = 1.37	*P* = 0.2493
P*Treat	*F* = 9.98	*P* = 0.0000	*F* = 23.43	*P* = 0.0000	*F* = 3.52	*P* = 0.0154	*F* = 1.58	*P* = 0.1984
Env*Treat	*F* = 13.43	*P* = 0.0000	*F* = 61.42	*P* = 0.0000	*F* = 9.84	*P* = 0.0000	*F* = 5.87	*P* = 0.0009
P*Env*Treat	*F* = 5.61	*P* = 0.0012	*F* = 19.65	*P* = 0.0000	*F* = 0.25	*P* = 0.9093	*F* = 7.19	*P* = 0.0002
CV	9.24		6.27		15.35		4.21	

### Rhizosphere pH

**FIGURE 12:**
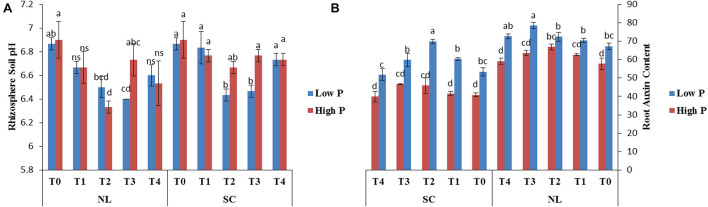
Effect of Ti application on **(A)** rhizosphere soil pH and **(B)** root Auxin content of soybean under NL and SC combined with LP and HP conditions. The bar plots with different lettering show the significant difference among cultivars at probability < 0.05. The error bars are the ± SD values with *n* = 3.

Under NL, no significant difference in rhizosphere pH values was observed in response to Ti treatments in HP and LP against T0, except for treatment T2 in HP and T3 in LP which led to a significant reduction (9.52 and 7.34%, respectively) in rhizosphere pH in comparison to T0. Overall, together under NL and SC, maximum (6.9) and minimum (6.33) rhizosphere pH was recorded in T0HP and T2HP, respectively in NL (Figure 12A). Table 6 shows the possible interaction of environment (SC, NL), Phosphorous treatments (LP, HP) in combination with Ti application for Rhizosphere pH.

**TABLE 6:** Values under ANOVA are the F-test, probabilities (*P* value) and coefficient of variation of the sources of variation (LSD, *P* < 0.05).

**ANOVA**				

**Factors**	**Rhizosphere pH**		**Root Auxin**	
Phosphorus (P)	*F* = 5.25	*P* = 0.0276	*F* = 458.74	*P* = 0.0000
Environment (Env)	*F* = 12.23	*P* = 0.0012	*F* = 685.15	*P* = 0.0000
Treatments (Treat)	*F* = 23.78	*P* = 0.0000	*F* = 31.61	*P* = 0.0000
P*Env	*F* = 1.76	*P* = 0.1927	*F* = 22.33	*P* = 0.0000
P*Treat	*F* = 5.54	*P* = 0.0013	*F* = 1.05	*P* = 0.3935
Env*Treat	*F* = 1.25	*P* = 0.3064	*F* = 5.14	*P* = 0.0021
P*Env*Treat	*F* = 2.34	*P* = 0.0724	*F* = 10.16	*P* = 0.0000
CV	1.61		4.04	

### Root Auxin Content

Under NL, T2 and T3 significantly increased (13 and 14%) the root Auxin content in comparison to control in HP and LP, respectively while the difference of other treatments with T0 remained non-significant. Overall, under NL, maximum (78.55) and minimum (57.67) values were recorded in T3LP and T0HP, respectively. Under SC, in HP, T2 and T3 led to a slight increase in root Auxin content as compared to control, though the increase was non-significant. In LP, T2 led to a significant increase (23.56%) in root Auxin content against T0 while the difference of other treatments with T0 remained non-significant. Overall, under SC, maximum (69.91) and minimum (59.20) values were recorded in T2LP and T4HP, respectively (Figure 12B). Table 6 shows the possible interaction of Environment (SC, NL), phosphorous treatments (LP, HP) in combination with Ti application for root Auxin content.

### Shoot P

**FIGURE 13:**
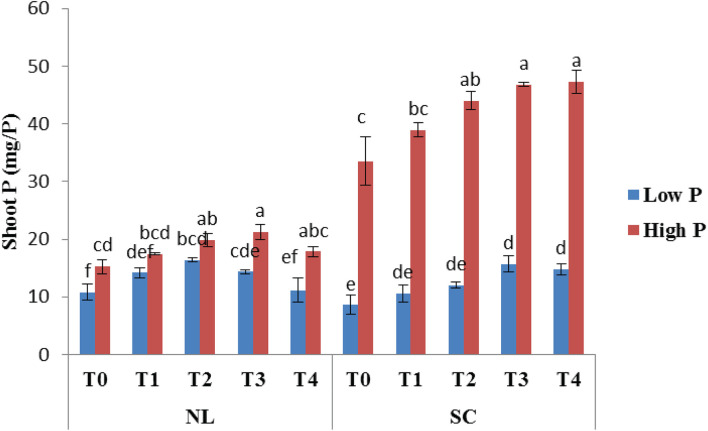
Effect of Ti application on shoot P content of soybean under NL and SC combined with LP and HP conditions. The bar plots with different lettering show the significant difference among cultivars at probability < 0.05. The error bars are the ± SD values with *n* = 3.

Under NL, T2 and T3 led to a significant increase in shoot P as compared to T0 in HP (23.25 and 28.27%) and LP (28.45 and 23.54%), respectively. Overall, maximum shoot P (21.26 mg/P) under NL was recorded in T3HP while minimum value (10.79 mg/P) was recorded in T0LP. Under SC, shoot P was significantly higher in HP as compared to LP. More specifically, T2, T3, and T4 led to a significant increase (23.85, 28.36, and 29.14% respectively) in shoot P against T0 in HP whereas in LP, T3 and T4 increased (45.29 and 41.93%, respectively) the shoot P significantly in comparison to control (Figure 13). Table 7 shows the possible interaction of environment (SC, NL), Phosphorous treatments (LP, HP) in combination with Ti application for shoot P content.

**TABLE 7:** Values under ANOVA are the F-test, probabilities (*P* value) and coefficient of variation of the sources of variation (LSD, *P* < 0.05).

**ANOVA**		

**Factors**	**Shoot P**	
Phosphorus (P)	*F* = 1511.77	*P* = 0.0000
Environment (Env)	*F* = 649.81	*P* = 0.0000
Treatments (Treat)	*F* = 34.74	*P* = 0.0000
P*Env	*F* = 771.28	*P* = 0.0000
P*Treat	*F* = 4.31	*P* = 0.0056
Env*Treat	*F* = 12.65	*P* = 0.0000
P*Env*Treat	*F* = 2.11	*P* = 0.0981
CV	8.04	

**FIGURE 14:**
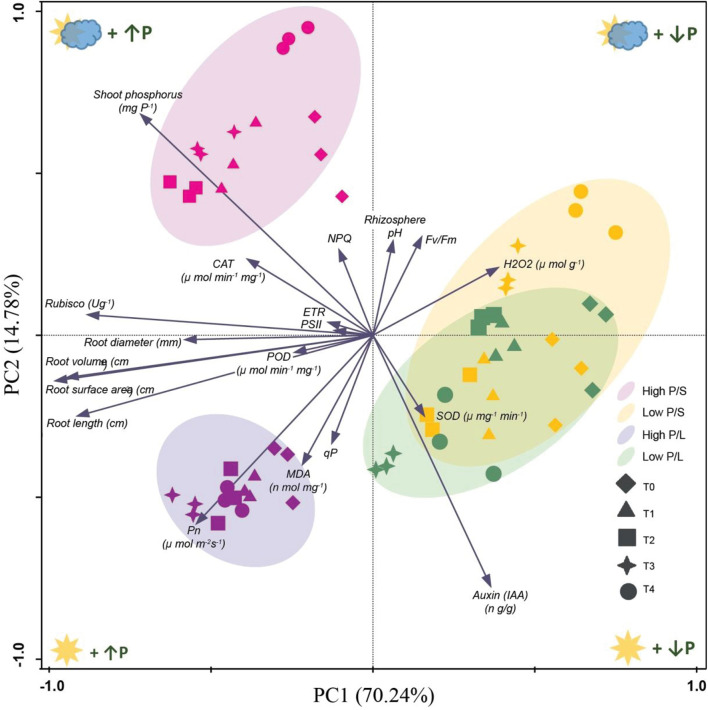
Principal component analysis (PCA) representing reaction of application of titanium on soybeans under normal light (NL) and shade conditions (SC) combined with low and high phosphorus (LP and HP) stress respectively. The first two axes (show in the diagram) explain 85% of the total variation.

In all variants, the positive effect of Ti application on the root architecture can be seen, compared to variants where Ti was not applied (diamonds). Ti application can partially compensate for the lack of phosphorus (LP). It can be seen that the controls are a little different from the others, which is not the case with the shade variant and a low P. Principal component analysis indicated that all the treatments in low P/S and low P/L were separated from high P/S and high P/L (Figure 14). The SOD activity was correlated with the T2 and T4 treatment under low P/S and low P/L, respectively. Whereas, hydrogen peroxide was related with T3 under low P/S. Pn activities were related with T1, T2, T3, and T4 under high P/L stress. Similarly, shoot phosphorus was correlated with T1, T2, and T3 treatments in high P/S. A greater amount of Auxin was observed in the illuminated variants than in the shady variants independent of the amount of phosphorus. Ti application improves phosphorus uptake. The role of Auxin in hypocotyl growth is described in (Ding et al., 2011).

## Discussion

Phosphorus being an essential macronutrient has a major role in crop production globally. In addition to the use of synthetic fertilizers, several other strategies have been recommended to mitigate the phosphorus deficiency-induced growth inhibition in plants (Wang et al., 2020). More specifically, the exo-application of elements and growth regulators as a source for agronomic adjustment has revealed favorable results in nutrient dearth soils. Ti is the 9^th^ most abundant element in the earth crust which is available to the plants from the rhizosphere soil solution. However, the role of Ti in plant biology, specifically its application under low light and phosphorus stress, has never been reported. In the current study, our findings reveal that foliar application of Ti not only increased its uptake to the aerial parts of the plant but also enhanced the shoot P content in soybean plants under low light and low phosphorus stress (Figure 13). Ti application and P uptake led to significant physiological variations (*P* < 0.05) as exhibited by root Auxin content, photosynthetic activity, efficiency of PSII, decreased ROS stress, and improved rhizosphere pH under shading and low P stress conditions. Our findings suggested that Ti application can alleviate shading and low P stress in dicotyledonous crops, such as soybean, and could be a useful agronomic approach to grow/raise/cultivate such crops in P dearth soils.

It is well known that Ti-induced resistance to abiotic stresses is facilitated by the inflection of endogenous hormones that somehow (directly or indirectly) activate or regulate different physiological changes like photosynthesis (Hussain et al., 2019a). For instance, Ti application enhances the efficiency of photosystem II and photosynthetic activity (Rubisco) in tomato (Haghighi and da Silva, 2014) and spinach (Zheng et al., 2005), which directly results in biomass increase, as revealed by enhanced stem diameter, plant height and root growth, suggesting a distinct effect of Ti on dry matter accumulation components. In the current study, shade and low phosphorus stress significantly decreased the net photosynthetic rate, which was associated with reduced efficiency of PSII in soybean leaves (Figure 6A). In addition, shade and low phosphorus stress may lead to disruption of phospholipids in chloroplast to release Pi, which could be a supportive evidence for decreased photosynthesis. Former studies reported that increased carbohydrates in chloroplast under low P conditions might result in reduced photosynthesis. However, foliar application of Ti significantly enhanced the Rubisco enzymatic activity and net photosynthetic rate under shade and low P stress as compared to low P without Ti application.

In support of our findings, previous studies reported that reverse transcription-polymerase chain reaction of Ti treated plants showed the increased expression of ribulose-1, 5-bisphosphate carboxylase/oxygenase (Rubisco) small subunit (rbcS), and Rubisco large subunit (rbcL) messenger RNA (mRNA) (Tumburu et al., 2015).

The generation of ROS is a natural and deliberated incidence in living organisms (plants) cells (Huang et al., 2019). However, the excessive production of ROS is known as a stress marker under biotic and abiotic stress conditions as ROS oxidizes the major biomolecules including proteins, lipids, and DNA (Farmer and Mueller, 2013). Oxidation of lipids can be quantified by malondialdehyde (MDA) content, which is an indication of oxidative stress. Generally, low light stress and a dearth of nutrient supply led to oxidative stress by excessive generation of ROS (Foyer and Shigeoka, 2011). In our present study, shading and low P conditions boosted oxidative stress as exhibited by enhanced MDA content (Figure 7D). The interactions of the treatments regarding MDA content were highly significant (*P* < 0.05) (Table 4). Therefore, it is imperative to scavenge ROS by moderating antioxidant enzymatic activities facilitating to curb the oxidative stress and boost nutrient uptake. Foliar application of Ti mitigate the oxidative stress in several plant species such as Moldavian balm (*Dracocephalummoldavica* L.) (Gohari et al., 2020), broad bean (Abdel Latef et al., 2018), and tomato (Raliya et al., 2015). Based on this, present research reveals that foliar application of Ti significantly alleviates the shading and low P induced oxidative stress as indicated by reduced ROS and MDA accumulation (Figure 7). This phenomenon of Ti-induced improvement was revealed more closely in the antioxidant’s activity of enzymes, such as CAT, POD, and SOD. Previous studies regarding Ti application either foliar or root treatment reported that Ti application increases the antioxidant enzymatic activities under UV light, salinity stress (Khan, 2016; Shah et al., 2021). In another study application of Ti enhances the generation of SOD, POD and CAT which potentially mitigates oxidative stress and improves salinity tolerance in broad beans (Abdel Latef et al., 2018). All these findings suggest that foliar application of Ti has the potential to regulate the antioxidants that can play a role to quench ROS produced in shade and low p stress in soybean plants.

Phosphorus (P) being macronutrient not only plays an important role in plant growth but also influences plant responses because of its direct association with ATP production and signaling by facilitating phosphorylation of several proteins (Maruyama and Wasaki, 2017). Thus, P dearth leads to metabolic and physiological changes in plants (Fukushima et al., 2017; Martínez-Andújar et al., 2017). As this study is remarkably novel, limited literature is available addressing the underlying mechanism of Ti-P uptake. Furthermore, this is the ever first study of Ti regarding combined phosphorus and the shade stress environment. However, few studies reported Ti application increases the nutrient uptake by affecting the root growth. In our present study, the decrease in rhizosphere pH (basic to acid) and root length and root surface area indicated the increased phosphorus uptake. In support of our finding, recently it is reported that Ti application significantly decreases the rhizosphere soil pH (basic to acidic) and enhances P uptake through increasing exudation of carboxylates ions in the rhizosphere soil (Zahra et al., 2015). In consistent with this, in our study Ti application under shade condition combined with low P might have activated the root exudates (malic acid, acetic acid and proton ions) and low phosphorus responsive genes.

The increase in root growth traits such as root hairs, root length, lateral root formation and root surface area is associated with root Auxin content (Alarcón et al., 2019; Guan et al., 2019). This increase in the morphological characteristics of roots due to the Auxin content significantly improves the efficiency of nutrient absorption, for example P. Previous study reported that low P and shade stress significantly reduces root length, root surface and root volume which ultimately leads to reduced nutrient uptake (Zhou et al., 2019). However, the application of Ti under low p and shade conditions significantly (*P* < 0.05) improved the root architecture (Figures 9–11) and root Auxin content (Figure 12B). The interaction analysis also confirmed the significant effect of all treatments (Tables 5, 6). In support of our findings, there are minuscule reports about the effect of Ti application on endogenous phytohormones under stress conditions, such as Arabidopsis root elongation and Auxin regulation. In this study, it was found that Ti promotes root elongation in Arabidopsis. After exposure to Ti, GUS expression in the DR5:GUS line was increased, whereas the fluorescent signal in DII-VENUS in root tips was reduced, indicating enhanced Auxin accumulation (Wei et al., 2020).

## Conclusion

In conclusion, our findings confirm that foliar application of Ti compensates the low phosphorus and shade stress-induced inhibited growth by improving root architectural traits, rhizosphere soil pH, root Auxin content, photosynthetic capacity, antioxidants, and phosphorus uptake in soybean. These results may practically be helpful in agronomic management of crops under P dearth and cereal legumes intercropping cultivating soils. Further investigation regarding root hairs and root exudates such as acetic acid, malic acid, and proton ions would further confirm the uptake of phosphorus due to Ti application. The genetic expression of low P responsive genes under Ti application needs to be investigated.

**FIGURE 15:**
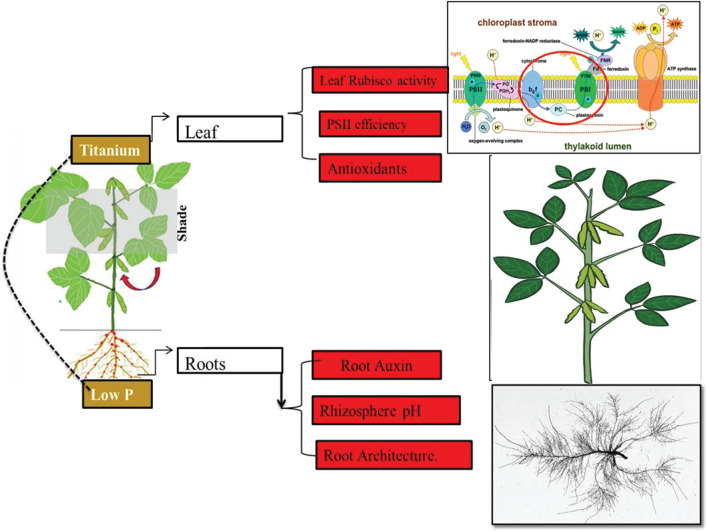
Mechanisms for increase in P uptake after Ti application.

Ti application can increase the P uptake by two-way mechanism (Figure 15). Above ground indication like an increase in leaf Rubisco activity and the efficiency of PSII confirmed the improved carbon metabolism. Below ground indications like improved root architecture, rhizosphere acidic pH and root Auxin content. A model showing a potential mechanism for Ti-induced amelioration of shade and low phosphorus stress is given below.

## Data Availability Statement

The original contributions presented in the study are included in the article/supplementary material, further inquiries can be directed to the corresponding author/s.

## Author Contributions

SH: design and conceived research and writing – original draft. IS, MB, AR, and MH: reviewing and editing. MS: PCA analysis editing. MM, NI, and MR: data curation. WL: project administration and supervision. WY: reviewing and supervision. All authors read and approved the manuscript.

## Conflict of Interest

The authors declare that the research was conducted in the absence of any commercial or financial relationships that could be construed as a potential conflict of interest.

## Publisher’s Note

All claims expressed in this article are solely those of the authors and do not necessarily represent those of their affiliated organizations, or those of the publisher, the editors and the reviewers. Any product that may be evaluated in this article, or claim that may be made by its manufacturer, is not guaranteed or endorsed by the publisher.
